# Generation of a Quantitative Luciferase Reporter for Sox9 SUMOylation

**DOI:** 10.3390/ijms21041274

**Published:** 2020-02-13

**Authors:** Hideka Saotome, Atsumi Ito, Atsushi Kubo, Masafumi Inui

**Affiliations:** 1 Laboratory of Animal Regeneration Systemology, Department of Life Sciences, School of Agriculture, Meiji University, Kanagawa 214-8571, Japan; 2 Department of Developmental Neurobiology, Institute of Development, Aging and Cancer, Tohoku University, 4-1 Seiryo, Aoba, Sendai, Miyagi 980-8575, Japan; 3 Meiji University International Institute for Bio-Resource Research, Kanagawa 214-8571, Japan

**Keywords:** Sox9, SUMO, NanoBiT, PTM, cartilage, chondrocyte

## Abstract

Sox9 is a master transcription factor for chondrogenesis, which is essential for chondrocyte proliferation, differentiation, and maintenance. Sox9 activity is regulated by multiple layers, including post-translational modifications, such as SUMOylation. A detection method for visualizing the SUMOylation in live cells is required to fully understand the role of Sox9 SUMOylation. In this study, we generated a quantitative reporter for Sox9 SUMOylation that is based on the NanoBiT system. The simultaneous expression of Sox9 and SUMO1 constructs that are conjugated with NanoBiT fragments in HEK293T cells induced luciferase activity in SUMOylation target residue of Sox9-dependent manner. Furthermore, the reporter signal could be detected from both cell lysates and live cells. The signal level of our reporter responded to the co-expression of SUMOylation or deSUMOylation enzymes by several fold, showing dynamic potency of the reporter. The reporter was active in multiple cell types, including ATDC5 cells, which have chondrogenic potential. Finally, using this reporter, we revealed a extracellular signal conditions that can increase the amount of SUMOylated Sox9. In summary, we generated a novel reporter that was capable of quantitatively visualizing the Sox9-SUMOylation level in live cells. This reporter will be useful for understanding the dynamism of Sox9 regulation during chondrogenesis.

## 1. Introduction

Sox9 is a transcription factor that is crucial for chondrogenesis [1]. Sox9 promotes the proliferation, differentiation, and maintenance of chondrocytes by regulating its target genes. As such, its role is essential for embryonic cartilage formation [1,2]. *Sox9* acts in a dose-dependent manner, and its haploinsufficiency causes bone malformation and perinatal death in both mice and humans [3,4,5], while the overexpression of *Sox9* also causes abnormalities in skeletal elements [6]. Sox9 is also reported to have important roles in sex determination, pancreas formation, and tumorigenesis [7,8,9]. Importantly, pancreas differentiation also shows Sox9 dose dependency [8]. 

As maintaining an appropriate level of Sox9 activity is important for the developmental processes, it is controlled by many mechanisms, including SUMOylation [10,11,12]. SUMOylation is a post-translational modification (PTM), in which a small polypeptide, called Small Ubiquitin-like MOdifier (SUMO), is covalently conjugated to the substrate proteins [13]. The conjugation of SUMO induces changes in the protein structure, localization, or stabilization. The 398th lysine of Sox9 is SUMOylated in human cells (the 396th lysine of Sox9 in mice) [14,15,16]. Although SUMOylation represses the Sox9 activity in the neural crest cells of *Xenopus laevis* [17,18], its role in the chondrogenic process remains unclear. 

As SUMOylation might be involved in Sox9 dose regulation, its quantitative detection during chondrogenesis would give us deeper insight into this process. However, the quantitative and dynamic detection of SUMOylation of specific proteins is technically difficult. Thus far, no specific antibodies against SUMOylated protein have been reported, and SUMOylated proteins are usually detected by Western blotting, based on the shift in the molecular weight or by the combination of immunoprecipitation and Western blotting [15,18]. Since these methods are laborious, non-quantitative, and cannot be applied to live cells, a novel approach is required to detect dynamic changes in protein SUMOylation in live cells.

**Figure 1:**
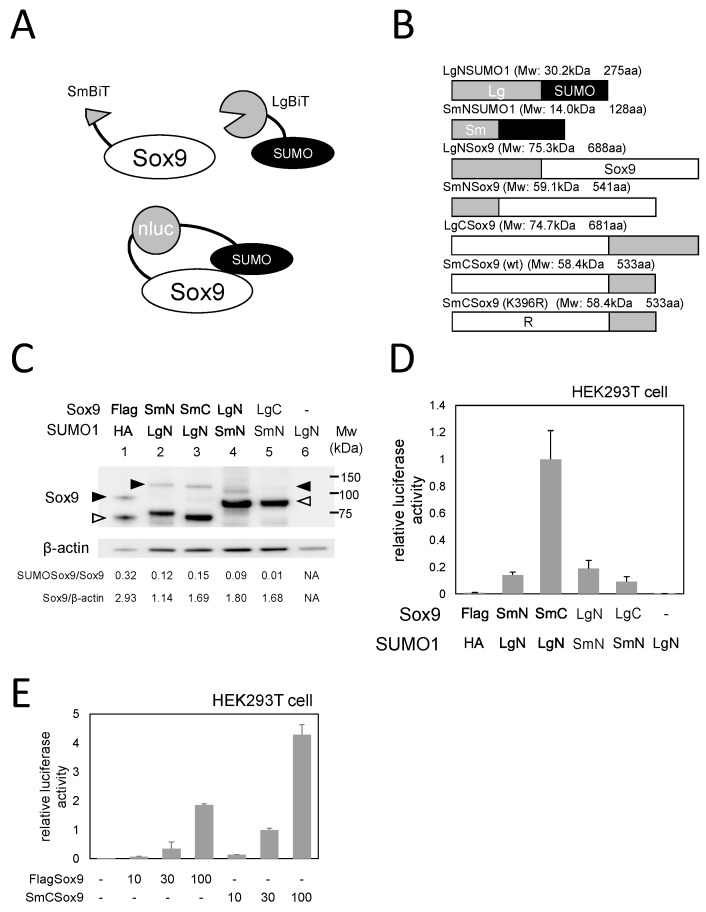
Generation of Small Ubiquitin-like Modifier-Sox9 (SUMO-Sox9) reporter. (**A**) Schematic diagrams of the NanoBiT reporter for detecting Sox9 SUMOylation. The upper figure indicates fusion proteins of NanoBiT fragments and Sox9 or SUMO1. The lower figure shows that the conjunction of Sox9 and SUMO1 causes the approach of SmBiT and LgBiT and, in turn, luciferase activity. (**B**) Seven types of NanoBiT fusion Sox9 and SUMO1. Gray bars indicate NanoBiT fragments. Black bars indicate SUMO1. White bars indicate Sox9. “R” in 7th construct indicates a K396R mutation. (**C**) Western blotting to investigate the expression and SUMOylation of NanoBiT fusion SUMO1 and Sox9. β-actin served as a loading control. White arrowheads indicate non-SUMOylated Sox9 and black arrowheads indicate SUMOylated Sox9. The ratios of SUMOylated/non-SUMOylated Sox9 and non-SUMOylated Sox9/ß-actin are shown at the bottom. NA indicates that one or both protein bands was not detected. (**D**) A luciferase assay to detect the reporter activity of NanoBiT fusion SUMO1 and Sox9. Bars indicate the mean value of triplicate wells. Error bars indicate the standard deviation. (**E**) A luciferase assay to detect the transcriptional activity of wild-type and SmCSox9. Bars indicate the mean value of triplicate wells. Error bars indicate the standard deviation. All of the experiments were repeated at least twice, and representative results are shown.

NanoBiT is a luciferase-based system for detecting protein-protein interaction by the complementation of separated NanoLuc fragments [19]. These fragments—known as LgBiT and SmBiT—have a low affinity for each other, so they only exert luciferase activity when their combined proteins interact [19]. This reporter is often used for assessing non-covalent protein interactions, but it can also be applied to detect protein PTMs [20]. We used this system to generate a quantitative reporter for Sox9 SUMOylation that could be applied to live cells (Figure 1A).

## 2. Results

### 2.1. Generation of a NanoBiT reporter for Sox9 SUMOylation

First, we cloned fusion constructs for SUMO1/Sox9 and NanoBiT fragments (*i.e.*, LgBiT or SmBiT). As the C-terminal of SUMO1 could not be modified, the fragments were fused to the N/C terminal of Sox9 and the N terminal of SUMO1 (Figure 1B, Supplementary Information 1). These constructs were introduced into HEK293T cells in all possible combinations, in order to examine whether or not the NanoBiT fusion Sox9 and SUMO1 protein could be conjugated by the endogenous SUMOylation machinery in the cells. The Flag-Sox9 and HA-SUMO1 constructs were also transfected to serve as a positive control. 

Western blotting with a Sox9 antibody visualized non-SUMOylated Flag-Sox9 (Figure 1C lane 1 white arrowhead) and SUMOylated Flag-Sox9 (Figure 1C lane 1 black arrowhead). The expression of NanoBiT fusion Sox9 constructs was detected with the expected molecular weight (Figure 1C lanes 2–5, white arrowhead). Furthermore, the SUMOylated form of Sox9 was also detected with the co-expression of NanoBiT fusion constructs (Figure 1C lane 2–5 black arrowhead). The bands of SUMOylated Sox9, Non-SUMOylated Sox9, and ß-actin (loading control) in each sample were quantified by densitometry and the ratio of the proteins was shown at the bottom of the panel.

Next, we performed a luciferase assay using the cell lysate of HEK293T cells in which the same combinations of NanoBiT fusion Sox9 and SUMO1 were expressed to investigate whether the conjunction of NanoBiT fusion SUMO1 and Sox9 produced luciferase activity. Although the expression of LgNSUMO1 alone was not associated with detectable luciferase activity, a clear luciferase signal was detected with all of the combinations of NanoBiT fusion SUMO1 and Sox9 (Figure 1D). In particular, the combination of SmCSox9 and LgNSUMO1 yielded the highest reporter activity (Figure 1D). These results indicated that we successfully generated a NanoBiT reporter for Sox9 SUMOylation, and the combination of SmCSox9/LgNSUMO1 is subsequently referred to as the “SUMO-Sox9 reporter” in this report. We also confirmed that the conjugation of the SmBiT fragment to its C-terminus did not affect the characteristics of Sox9 as a transcription factor. SmCSox9 activated the luciferase reporter activity similarly to the wild type Sox9 on *Collagen type II* promoter luciferase (Figure 1E).

Of note, the ratio of SUMOylated/non-SUMOylated Sox9 with NanoBiT fusion constructs was lower than that with wild-type Sox9, probably due to the fusion of NanoBiT fragments. Nonetheless, a low SUMOylation ratio does not compromise the usefulness of the reporter, as the purpose of this reporter is not to detect the absolute amount or ratio of Sox9 SUMOylation, but rather to detect dynamic changes (i.e., upregulation or downregulation) in SUMOylation in response to various cellular contexts, such as cell differentiation.

### 2.2. SUMO-Sox9 Reporter Activity Depends on Sox9 SUMOylation and Is Quantitatively Dynamic

**Figure 2:**
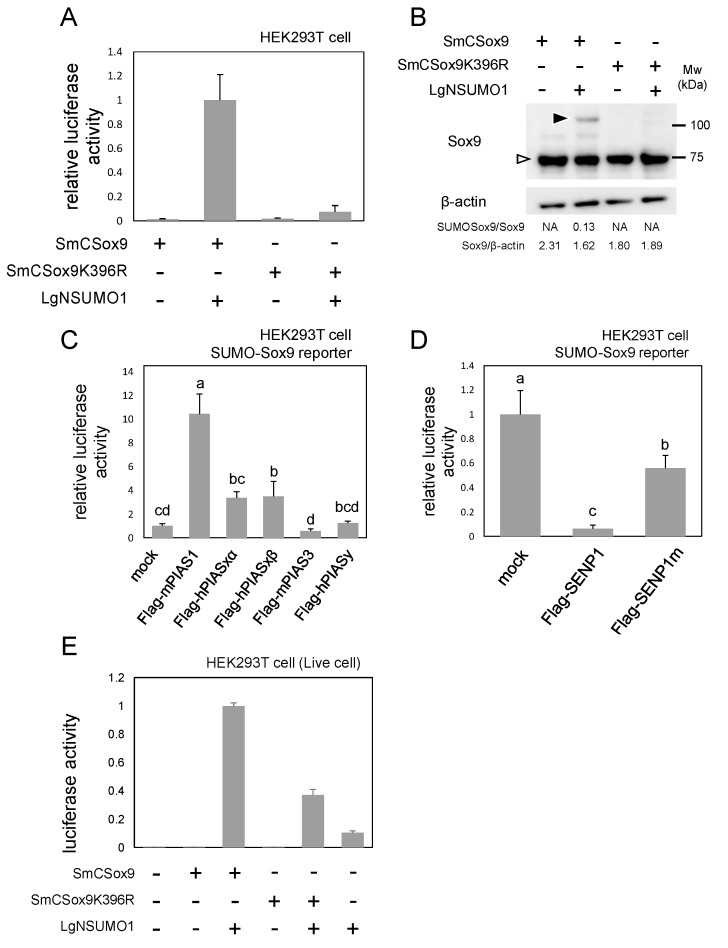
SUMO-Sox9 reporter activity in HEK293T cells. (**A**) A luciferase assay to examine the SUMOylation dependency of SUMO-Sox9 reporter. Bars indicate the mean value of triplicate wells. Error bars indicate the standard deviation. (**B**) Western blotting of the cell extract prepared as those of (**A**). White arrowhead indicates Sox9. Black arrowhead indicates SUMOylated Sox9. β-actin served as a loading control. The ratios of SUMOylated/non-SUMOylated Sox9 and non-SUMOylated Sox9/ß-actin are shown at the bottom. NA indicates that one or both protein bands were not detected. (**C**) A luciferase assay to examine whether or not the SUMO-Sox9 reporter activity could be upregulated with the promotion of Sox9 SUMOylation. Bars indicate the mean value of triplicate wells. Error bars indicate the standard deviation. The mean values sharing a letter did not differ to a statistically significant extent (*p* ≥ 0.05, Tukey’s HSD test). (**D**) A luciferase assay to examine whether or not the SUMO-Sox9 reporter activity could be downregulated with Sox9 deSUMOylation. The bars indicate the mean value of triplicate wells. Error bars indicate the standard deviation. The mean values sharing a letter did not differ to a statistically significant extent (*p* ≥ 0.05, Tukey’s HSD test). (**E**) A luciferase assay to detect the SUMO-Sox9 reporter activity from live cells. Bars indicate the mean value of triplicate wells. Error bars indicate the standard deviation. All experiments were repeated at least twice, and representative results are shown.

We carried out the same luciferase assay using SmCSox9 with a K-to-R mutation in its SUMOylation target (lysine 396) (SmCSox9K396R, Figure 1B) in order to confirm whether or not SUMO-Sox9 reporter activity depends truly on Sox9 SUMOylation. The results showed that the SUMO-Sox9 reporter activity was markedly lower with SmCSox9K396R than with wild-type SmCSox9 (Figure 2A), which was consistent with the results of Western blotting, where SUMOylation was not detected with SmCSox9K396R (Figure 2B). These results indicate that the SUMO-Sox9 reporter activity depends on Sox9 SUMOylation. 

In addition, we co-expressed SUMOylation enzymes in HEK293T cells with SUMO-Sox9 reporter to check whether or not SUMO-Sox9 reporter could quantitatively reflect the change in the Sox9 SUMOylation level. The co-expression of PIAS1 and PIASxβ increased the reporter activity, whereas PIASy and PIAS3 had little effect (Figure 2C). The co-expression of PIASxα also increased the reporter activity, although the increase was not statistically significant. Conversely, the co-expression of deSUMOylation enzyme SENP1 repressed the reporter activity (Figure 2D). The co-expression of SENP1m, an SENP1 enzyme-dead mutant also had a weak, but significant, repressing effect, which implies that enzyme activity-independent repressing activity might also exist (Figure 2D). The SUMO-Sox9 reporter activity could also be detected from live cells, and this activity was decreased by the inhibition of SUMOylation using SmCSox9K396R (Figure 2E). Therefore, we confirmed that, the SUMO-Sox9 reporter activity is SUMOylation-dependent and that it can visualize Sox9 SUMOylation quantitatively in both cell lysates and live cells in HEK293T cells.

### 2.3. SUMO-Sox9 Reporter Is Active also in Other Cell Lines

**Figure 3:**
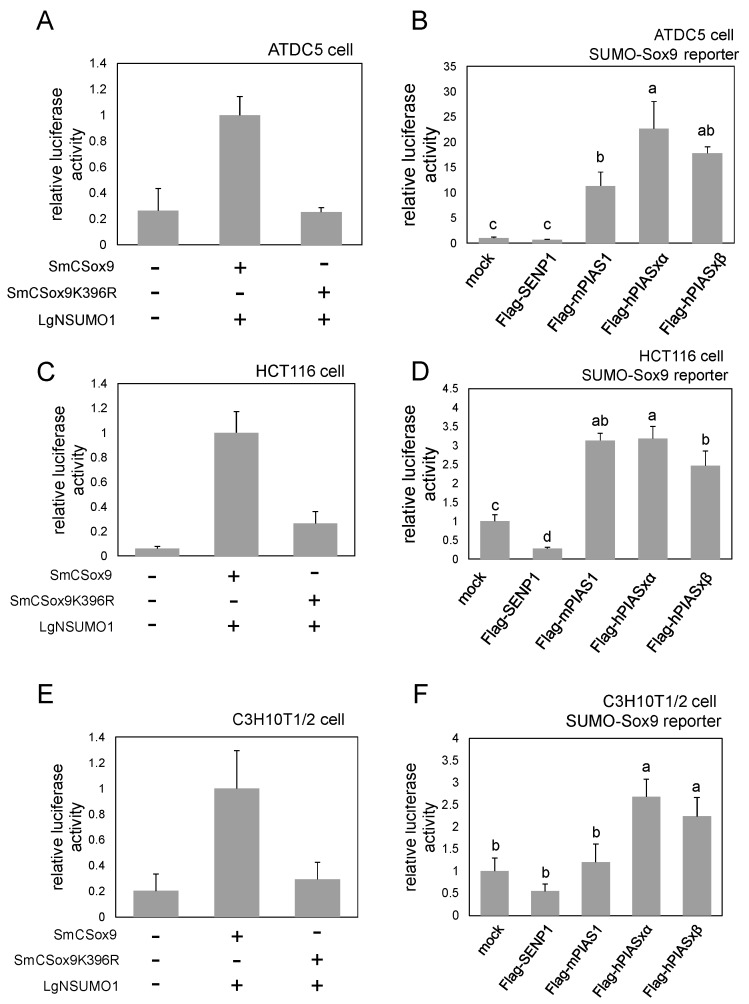
SUMO-Sox9 reporter activity in ATDC5, HCT116 and C3H10T1/2 cells. (**A**) A luciferase assay to examine the SUMOylation-dependent activity of SUMO-Sox9 reporter in ATDC5 cells. Bars indicate the mean value of triplicate wells. Error bars indicate the standard deviation. (**B**) A luciferase assay to examine whether or not the SUMO-Sox9 reporter activity could be upregulated with the promotion of Sox9 SUMOylation and downregulated with the Sox9 deSUMOylation in ATDC5 cells. Bars indicate the mean value of triplicate wells. Error bars indicate the standard deviation. The mean values sharing a letter did not differ to a statistically significant extent (*p* ≥ 0.05, Tukey’s HSD test). (**C**) A luciferase assay to examine the SUMOylation-dependent activity of SUMO-Sox9 reporter in HCT116 cells. Bars indicate the mean value of triplicate wells. Error bars indicate the standard deviation. (**D**) A luciferase assay to examine whether or not the SUMO-Sox9 reporter activity could be upregulated with the promotion of Sox9 SUMOylation and downregulated with Sox9 deSUMOylation in HCT116 cells. Bars indicate the mean value of triplicate wells. Error bars indicate the standard deviation. The mean values sharing a letter did not differ to a statistically significant extent (*p* ≥ 0.05, Tukey’s HSD test). (**E**) A luciferase assay to examine the SUMOylation-dependent activity of SUMO-Sox9 reporter in C3H10T1/2 cells. Bars indicate the mean value of triplicate wells. Error bars indicate the standard deviation. (**F**) A luciferase assay to examine whether or not the SUMO-Sox9 reporter activity could be upregulated with the promotion of Sox9 SUMOylation and downregulated with the Sox9 deSUMOylation in C3H10T1/2 cells. Bars indicate the mean value of triplicate wells. Error bars indicate the standard deviation. The mean values sharing a letter did not differ to a statistically significant extent (*p* ≥ 0.05, Tukey’s HSD test). All of the experiments were repeated at least twice, and representative results are shown.

We expressed this reporter in several other cell lines that are more biologically relevant for the Sox9 activity to examine whether or not SUMO-Sox9 reporter can be used in other cell lines. Namely, ATDC5 cells that have the potential to differentiate into chondrocytes, HCT116 cells derived from colon cancer, and C3H10T1/2 cells established from embryonic fibroblasts were examined. As in HEK293T cells, SUMO-Sox9 reporter showed luciferase activity in a K396-dependent manner in all three cell types (Figure 3A,C,E). In addition, the co-expression of PIAS proteins elevated the reporter activity (Figure 3B,D,F). The co-expression of SENP1 repressed the reporter activity in HCT116 cells, but it had less effect in ATDC5 and C3H10T1/2 cells (Figure 3B,D,F). In summary, our results showed that SUMO-Sox9 reporter could also be applied to other cell types and implied that the effect of Sox9 SUMOylation/deSUMOylation enzymes might differ among cell types, possibly depending on the endogenous SUMOylation level or other factors.

### 2.4. SUMO-Sox9 Reporter Revealed that FGF Signal Increases the Amount of SUMOylated Sox9

**Figure 4:**
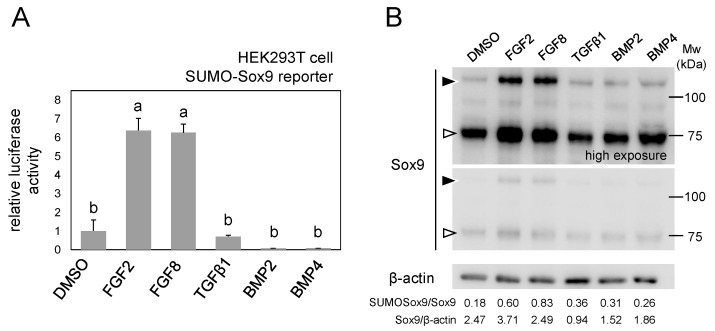
FGF signaling increases SUMOylated Sox9. (**A**) A luciferase assay for detecting the effects of cytokine treatments on Sox9 SUMOylation. HEK293T cells were transfected with SUMO-Sox9 reporter and treated with cytokines for 18 h, and the luciferase activity was then measured. Bars indicate the mean value of triplicate wells. Error bars indicate the standard deviation. The mean values sharing a letter did not differ to a statistically significant extent (*p* ≥ 0.05, Tukey’s HSD test). (**B**) Western blotting of the cell extract prepared as those in (**A**). Sox9 blots with shorter exposure (middle panel) and longer exposure (upper panel) are shown. β-actin served as a loading control. White arrowhead indicates Sox9, and black arrowhead indicates SUMOylated Sox9. The ratios of SUMOylated/non-SUMOylated Sox9 and non-SUMOylated Sox9/ß-actin are shown at the bottom. All experiments were repeated at least twice, and representative results are shown.

We next wanted to use this reporter to reveal the cellular context in which the amount of SUMOylated Sox9 changes, as we confirmed that SUMO-Sox9 reporter was able to quantitatively detect dynamic changes in Sox9 SUMOylation. As an example, we used this reporter to detect changes in Sox9 SUMOylation upon the activation of several signal transduction pathways. Among the five types of cytokines that we tested, FGF2 and FGF8 treatment increased the SUMO-Sox9 reporter activity, while TGFβ, BMP2 and BMP4 had little or no effect (Figure 4A). We also examined these samples by Western blotting to validate the increase of the amount of SUMOylated Sox9 in FGF2- or FGF8-treated cells. The amounts of SUMOylated Sox9 of FGF2- or FGF8-treated samples were much higher in comparison to the DMSO-treated samples or samples that were treated with other cytokines (Figure 4B). The quantification of protein bands implied that FGF2/8 treatment also promoted the degree of Sox9 SUMOylation. We quantified the bands with shorter exposure to avoid the saturation of signals, as we noticed that the relative intensity of the bands of Sox9 or SUMOylated Sox9 varied substantially among samples (Figure 4B middle panel). Taken together, our reporter could identify extracellular signals that increase the amount of SUMOylated Sox9 in the cells.

## 3. Discussion

Sox9 is a critical transcription factor for chondrogenesis, and its activity changes dynamically during the process, but, at the same time, it is precisely regulated [1,2,3,4,5,6,7]. SUMOylation is reported to regulate the Sox9 activity in cultured cells [14,15,16]. As such, it is important to understand when and how much Sox9 is SUMOylated during chondrogenesis. However, Sox9 SUMOylation is conventionally detected by Western blotting, which makes it difficult to examine quantitatively or to examine its temporal dynamics. In the present study, we generated a novel reporter based on the highly sensitive NanoBiT luciferase technology to solve these difficulties in detecting Sox9 SUMOylation. As Sox9 also has important roles in sex determination, pancreatic differentiation, and tumor formation, the quantification of Sox9 SUMOylation could impact these biological processes.

Our reporter signal faithfully reflected the Sox9 SUMOylation on K396 (Figure 2) and quantitatively responded to SUMOylation enzymes PIAS1 and PIASxβ and the deSUMOylation enzyme SENP1 (Figure 2). Furthermore, the reporter was also able to detect the SUMOylation level of Sox9 in live cells (Figure 2). Our SUMO-Sox9 reporter thus functioned as a sensitive and quantitative detection system for Sox9 SUMOylation. 

We found that the SUMOylation level of SmCSox9/LgNSUMO1 and SmNSox9/LgNSUMO1 was similar on Western blotting while examining the combinations of LgBiT/SmBiT conjugated SUMO1 and Sox9, but that the luciferase activity in the former was much higher than that in the latter (Figure 1). These results indicate that the reporter activity depends on not only the degree of SUMOylation, but also the mutual position of the NanoBiT fragments. 

FRET technology has also been used to quantitatively analyze protein SUMOylation [21,22]. FRET has an advantage in visualizing the SUMOylated protein with spatial information, such as subcellular localization, but the NanoBiT reporter has high sensitivity, so these two techniques can serve as alternative options, depending on the purpose of detection. Furthermore, the small size of NanoBiT fragments is also useful for conjugating with relatively small PTM molecules, as this approach has also been used to detect other PTMs, such as neddylation [20]. 

We confirmed that our SUMO-Sox9 reporter is also active in ATDC5 cells, which has the potential to differentiate into chondrocytes (Figure 3). It would be very interesting to observe the dynamics of Sox9 SUMOylation during the chondrocyte differentiation process in a future study, as the SUMO-Sox9 reporter was able to detect signal from live cells (Figure 2). Of note, the response to the co-expression of SUMOylation/deSUMOylation enzymes was not completely consistent among different cell lines. For example, PIAS1 was very efficient for promoting SUMOylation in HEK293T cells, but was not as effective in ATDC5 cells (Figure 3). Differences in the endogenous SUMOylation level or the expression of enzymes among cell types might underlie these observations. Similarly, PIAS3 was reported to SUMOylate Sox9 in an in vitro assay [16], but it did not alter the SUMO-Sox9 reporter in HEK293T cells in our study (Figure 2). These findings imply a context- and cell type-dependent manner of Sox9 SUMOylation, and our reporter will be useful for clarifying cellular contexts affecting Sox9 modification. In fact, we found that FGF treatment was able to increase the amount of SUMOylated Sox9 while using the SUMO-Sox9 reporter (Figure 4). It will be interesting to examine how SUMOylation is involved in the FGF-Sox9-chondrogenesis axis, as FGF signaling is known to regulate skeletal development through the Sox9 protein level [23]. In this study, we transiently expressed the reporter in cultured cell lines, and thus the observation was limited to a short period of time. Furthermore, standardization with a co-transfected reference, such as firefly luciferase, is required to correct the effect of transfection efficiency. Cells stably expressing this reporter, or a knock-in reporter animal model, would be ideal for the long term or in vivo observation of Sox9 SUMOylation in the future.

In conclusion, we have generated a novel quantitative reporter for Sox9 SUMOylation. Previous studies have revealed the role of Sox9 SUMOylation in neural crest cells [17,18], but how it is involved in other biological processes remains unclear. We previously generated mice with a K396R mutation in Sox9 and are currently investigating its effect on skeletal development [24]. We believe that this reporter can be useful for revealing the function of Sox9 SUMOylation in various processes, which include chondrogenesis and skeletal development.

## 4. Materials and Methods 

### 4.1. Cell Culture

HEK293T, HCT116, and C3H10T1/2 cells were cultured in Dulbecco’s Modified Eagle Medium (DMEM; Thermo Fisher, Waltham, MA, USA) that was supplemented with 10% fetal bovine serum (FBS; Biowest, Nuaillé, France) and Penicillin-Streptomycin-Glutamine mixed solution (Nacalai Tesque, Kyoto, Japan), and incubated at 37 °C under 5% CO_2_. ATDC5 cells were cultured in Alpha modification of Eagle’s MEM (α-MEM; Nacalai Tesque) supplemented with 5% FBS (Biowest), Penicillin-Streptomycin-Glutamine Mixed Solution (Nacalai Tesque) and Insulin-Transferrin-Selenium solution (ITS-G; Thermo Fisher), and then incubated at 37°C under 5% CO_2_.

### 4.2. Plasmid Construction

Sox9 (Sox9: NM_011448) and SUMO1 (Sumo1: NM_009460) were amplified from cDNA of E13.5 mouse testis cDNA and then inserted into pBiT1.1N and pBiT2.1N to make a fusion construct with Sm/LgBiT (Promega, Fitchburg, WI, USA). Sox9 subcloned into pBiT1.1C Sox9 and pBit2.1C were codon optimized while using a GeneArt GeneOptimizer and synthesized by gBlocks (IDT, Coralville, IA, USA) to facilitate the cloning process. The fusion constructs were subcloned into pcDNA3.1. pGL4.53 was purchased from Promega. Flag-mPIAS1 (ID:15206) [25], Flag-mPIAS3 (ID:15207) [26], Flag-hPIASy (ID:15208) [27], Flag-hPIASx alpha (ID:15209) [28], Flag-hPIASx beta (ID:15210) [28], and Flag-SENP1(ID:17357) [29] were purchased from Addgene (Wartertown, MA, USA). 

### 4.3. Western Blotting

The cells were lysed with lysis buffer (50 mM HEPES pH 7.8, 200 mM NaCl, 5 mM EDTA, 1% NP40, 5% Glycerol, 1 mM DTT, 20 mM N-ethylmaleimide,) with protease inhibitor cocktail (cOmplete; SIGMA-Aldrich, Saint Louis, MO, USA). Lysate was boiled at 95°C for 10 min. before sodium dodecyl sulfate-polyacrylamide gel electrophoresis (SDS-PAGE) to deactivate deSUMOylation enzymes. The amounts of each sample loaded for SDS-PAGE were calculated according to the co-transfected firefly luciferase signal to ensure the equal loading of transfected proteins. After SDS-PAGE, the samples were transferred to PVDF membranes (Immobilon-P; Merck, Kenilworth, NJ, USA) for immunoblotting. The primary antibodies used were α-Sox9 (AB5535, Merck, 1:1000) and α-β-actin (M177-3, MBL, Nagoya, Japan, 1:2000). The secondary antibodies used were α-rabbit IgG-Peroxidase (SIGMA-Aldrich, A0545, 1:2000) and α-mouse IgG-Peroxidase (SIGMA–Aldrich, A2304, 1:2000). Signal detection was performed with ECL Western Blotting Detection Reagents (GE Healthcare, Chicago, IL, USA), according to the manufacturer’s instructions. The proteins bands were quantified by densitometry using the ImageJ software program.

### 4.4. Luciferase Assay

HEK293T and HCT116 cells were transfected with the plasmids of NanoBiT fusion SUMO1 and Sox9 constructs (SUMO-Sox9 reporter), pGL4.53, SUMOylation enzymes, or deSUMOylation enzyme using FuGENE^®^ HD Transfection Reagent (Promega), according to the manufacturer’s protocols. C3H10T1/2 cells and ATDC5 cells were electroporated with plasmids of SUMO-Sox9 reporter, SUMOylation enzymes or deSUMOylation enzymes using the Neon^®^ Transfection System (Thermo Fisher), according to the manufacturer’s protocols. Forty-eight hours after transfection, the cells were lysed with the same lysis buffer, as was used for Western blotting, and the NanoBiT luciferase activity was measured while using a Nano-Glo^®^ Dual-Luciferase Reporter Assay System (Promega) in a 96-well plate. The nano-luc signals were standardized with firefly luciferase signal. The luciferase activities were shown as the mean value of triplicate wells + standard deviation. 

For the cytokine treatment experiment, cytokines were added 18 h before luciferase measurement. The following cytokines were used: 1 ng/mL TGFβ1 (PEPROTECH, Rocky Hill, NJ, USA; #100-21C), 100 ng/mL BMP2 (PEPROTECH; #120-02), 100 ng/mL BMP4 (R&D Systems, Minneapolis, MN, USA; #P21275), 100 ng/mL FGF2 (mouse) (CELL Guidance Systems, Cambridge, UK; GFM12), and 100 ng/mL FGF8 (R&D Systems; #NP_006110). The luciferase activity from live cells was detected using Nano-Glo^®^ Live Cell Assay System (Promega), by adding Nano-luc substrate to the culture media.

For the assay of Sox9 transcriptional activity, the HEK293T cells were transfected with the plasmids of *Col2a1* promoter luciferase reporter [30], Renilla luciferase reporter (pRL-SV40, Promega), and Flag-Sox9 or SmC-Sox9 plasmids using FuGENE^®^ HD Transfection Reagent (Promega), according to the manufacturer’s protocols. Forty-eight hours after transfection, the cells were lysed with the same lysis buffer as was used for Western blotting, and the firefly luciferase activity was measured using a Dual-Glo^®^ Luciferase assay kit (Promega). The firefly luciferase signals were standardized with Renilla luciferase signals.

### 4.5. Statistical Analysis

The differences among the luciferase activities were estimated using one-way analysis of variance (ANOVA), followed by Tukey’s test. The mean values sharing a letter in each luciferase panels did not differ to a statistically significant extent (*p* ≥ 0.05).

## Supplementary Materials

The following are available online at https://www.mdpi.com/1422-0067/21/4/1274/s1.
