# Supplementary material for: Generation of a Quantitative Luciferase Reporter for Sox9 SUMOylation
Source: Int J Mol Sci. 2020 Feb 13;21(4):1274. doi: 10.3390/ijms21041274 (PMC7072981; doi:10.3390/ijms21041274)
Supplement: Supplementary file 1 [file ijms-21-01274-s001.pdf]

## SmCSox9EX(Mw: 58.406kDa)

MNLLDPFMKMTDEQEKGLSGAPSPTMSEDSAGSPCPSGSGSDTENTRPQENTFPKGEPDLKKESEEDKFPVCI
REAVSQVLKGYDWTLVPMPVRVNGSSKNKPHVKRPMNAFMVWAQAARRKLADQYPHLHNAELSKTLGKLWRLL
NESEKRPFVEEAERLRVQHKKDHPDYKYQPRRRKSVKNGQAEAEEATEQTHISPNAIFKALQADSPHSSSGMS
EVHSPGEHSGQSQGPPTPPTTPKTDVQAGKVDLKREGRPLAEGGRQPPIDFRDVDIGELSSDVISNIETFDVN
EFDQYLPPNGHPGVPATHGQVTYTGSYGISSTAPTPATAGHVWMSKQQAPPPPPQQPPQAPQAPQAPPQQQAP
PQQPQAPQQQQAHTLTTLSSEPGQSQRTHIKTEQLSPSHYSEQQQHSPQQISYSPFNLPHYSPSYPPITRSQY
DYADHQNSGSYYSHAAGQGSGLYSTFTYMNPAQRPMYTPIADTSGVPSIPQTHSPQHWEQPVYTQLTRPGSSG
GGGSGGGGSSG**VTGYRLFEEIL***

## SmNSox9(Mw: 59.052kDa)

**MVTGYRLFEEIL**GSSGGGGSGGGGSSGGAQGNSMNLLDPFMKMTDEQEKGLSGAPSPTMSEDSAGSPCPSGSG
SDTENTRPQENTFPKGEPDLKKESEEDKFPVCIREAVSQVLKGYDWTLVPMPVRVNGSSKNKPHVKRPMNAFM
VWAQAARRKLADQYPHLHNAELSKTLGKLWRLLNESEKRPFVEEAERLRVQHKKDHPDYKYQPRRRKSVKNGQ
AEAEEATEQTHISPNAIFKALQADSPHSSSGMSEVHSPGEHSGQSQGPPTPPTTPKTDVQAGKVDLKREGRPL
AEGGRQPPIDFRDVDIGELSSDVISNIETFDVNEFDQYLPPNGHPGVPATHGQVTYTGSYGISSTAPTPATAG
HVWMSKQQAPPPPPQQPPQAPQAPQAPPQQQAPPQQPQAPQQQQAHTLTTLSSEPGQSQRTHIKTEQLSPSHY
SEQQQHSPQQISYSPFNLPHYSPSYPPITRSQYDYADHQNSGSYYSHAAGQGSGLYSTFTYMNPAQRPMYTPI
ADTSGVPSIPQTHSPQHWEQPVYTQLTRP*

## LgCSox9EX(Mw: 74.665kDa)

MNLLDPFMKMTDEQEKGLSGAPSPTMSEDSAGSPCPSGSGSDTENTRPQENTFPKGEPDLKKESEEDKFPVCI
REAVSQVLKGYDWTLVPMPVRVNGSSKNKPHVKRPMNAFMVWAQAARRKLADQYPHLHNAELSKTLGKLWRLL
NESEKRPFVEEAERLRVQHKKDHPDYKYQPRRRKSVKNGQAEAEEATEQTHISPNAIFKALQADSPHSSSGMS
EVHSPGEHSGQSQGPPTPPTTPKTDVQAGKVDLKREGRPLAEGGRQPPIDFRDVDIGELSSDVISNIETFDVN
EFDQYLPPNGHPGVPATHGQVTYTGSYGISSTAPTPATAGHVWMSKQQAPPPPPQQPPQAPQAPQAPPQQQAP
PQQPQAPQQQQAHTLTTLSSEPGQSQRTHIKTEQLSPSHYSEQQQHSPQQISYSPFNLPHYSPSYPPITRSQY
DYADHQNSGSYYSHAAGQGSGLYSTFTYMNPAQRPMYTPIADTSGVPSIPQTHSPQHWEQPVYTQLTRPGSSG
GGGSGGGGSSG**VFTLEDFVGDWEQTAAYNLDQVLEQGGVSSLLQNLAVSVTPIQRIVRSGENALKIDIHVIIP
YEGLSADQMAQIEEVFKVVYPVDDHHFKVILPYGTLVIDGVTPNMLNYFGRPYEGIAVFDGKKITVTGTLWNG
NKIIDERLITPDGSMLFRVTINS***

## LgNSox9(Mw: 75.311kDa)

**MVFTLEDFVGDWEQTAAYNLDQVLEQGGVSSLLQNLAVSVTPIQRIVRSGENALKIDIHVIIPYEGLSADQMA**
**QIEEVFKVVYPVDDHHFKVILPYGTLVIDGVTPNMLNYFGRPYEGIAVFDGKKITVTGTLWNGNKIIDERLIT**
**PDGSMLFRVTINS**GSSGGGGSGGGGSSGGAQGNSMNLLDPFMKMTDEQEKGLSGAPSPTMSEDSAGSPCPSGS
GSDTENTRPQENTFPKGEPDLKKESEEDKFPVCIREAVSQVLKGYDWTLVPMPVRVNGSSKNKPHVKRPMNAF
MVWAQAARRKLADQYPHLHNAELSKTLGKLWRLLNESEKRPFVEEAERLRVQHKKDHPDYKYQPRRRKSVKNG
QAEAEEATEQTHISPNAIFKALQADSPHSSSGMSEVHSPGEHSGQSQGPPTPPTTPKTDVQAGKVDLKREGRP
LAEGGRQPPIDFRDVDIGELSSDVISNIETFDVNEFDQYLPPNGHPGVPATHGQVTYTGSYGISSTAPTPATA
GHVWMSKQQAPPPPPQQPPQAPQAPQAPPQQQAPPQQPQAPQQQQAHTLTTLSSEPGQSQRTHIKTEQLSPSH
YSEQQQHSPQQISYSPFNLPHYSPSYPPITRSQYDYADHQNSGSYYSHAAGQGSGLYSTFTYMNPAQRPMYTP
IADTSGVPSIPQTHSPQHWEQPVYTQLTRP*

## SmNSUMO1(Mw: 13.959kDa)

**MVTGYRLFEEIL**GSSGGGGSGGGGSSMSDQEAKPSTEDLGDKKEGEYIKLKVIGQDSSEIHFKVKMTTHLKKL
KESYCQRQGVPMNSLRFLFEGQRIADNHTPKELGMEEEDVIEVYQEQTGGHSTV*

## LgNSUMO1(Mw: 30.218kDa)

**MVFTLEDFVGDWEQTAAYNLDQVLEQGGVSSLLQNLAVSVTPIQRIVRSGENALKIDIHVIIPYEGLSADQMA**
**QIEEVFKVVYPVDDHHFKVILPYGTLVIDGVTPNMLNYFGRPYEGIAVFDGKKITVTGTLWNGNKIIDERLIT**
**PDGSMLFRVTINS**GSSGGGGSGGGGSSMSDQEAKPSTEDLGDKKEGEYIKLKVIGQDSSEIHFKVKMTTHLKK
LKESYCQRQGVPMNSLRFLFEGQRIADNHTPKELGMEEEDVIEVYQEQTGGHSTV*

Supplemental information

Deduced amino acid sequences of NanoBiT fusion constructs which were shown in Figure 1B. Underlines indicate linker sequence and Bold characters indicate Sm/LgBiT sequence.
